# The influence of adding modified zirconium oxide-titanium dioxide nano-particles on mechanical properties of orthodontic adhesive: an in vitro study

**DOI:** 10.1186/s12903-017-0332-2

**Published:** 2017-01-13

**Authors:** Nayef H. Felemban, Mohamed I. Ebrahim

**Affiliations:** 1 Orthodontic department, Faculty of Dentistry, Taif University, Taif, Saudi Arabia; 2 Restorative dentistry department, Faculty of Dentistry, Taif University, Taif, Saudi Arabia; 3 P.O.B. 4302, Makkah, 21955 Saudi Arabia

**Keywords:** Orthodontic adhesives, Titanium dioxide, Zirconium oxide

## Abstract

**Background:**

The purpose of this in-vitro study was to examine the effect of incorporating different concentrations of Zirconium oxide-Titanium dioxide (ZrO2-TiO2) nanoparticles, which can have antibacterial properties, on the mechanical properties of an orthodontic adhesive.

**Methods:**

ZrO2-TiO2 (Zirconium oxide, HWNANO, Hongwu International Group Ltd, China) -Titanium dioxide, Nanoshell, USA) nanopowder were incorporated into orthodontic adhesive (Transbond XT, 3 M Unitek, Monrovia, USA) with different concentrations (0.5% weight nonofiller and 1% weight nanofiller). The size of nanoparticle was 70–80 nm for ZrO2 and less than 50 nm for TiO2. For measuring the shear bond strength of the three groups of orthodontic adhesives [Transbond (control), Transbond mixed with 0.5% weight ZrO2-TiO2, and Transbond mixed with 1% weight ZrO2-TiO2], 30 freshly extracted human first premolars were used and bonded with stainless steel metal brackets (Dentaurum®, Discovery®, Deutschland), using the 3 orthodontic adhesives and 3 M Unitek; Transbond TM Plus Self-Etching Primer (10 samples in each group). The recorded values of compressive strength and tensile strength (measured separately on 10 samples of orthodontic adhesives (add the 3 D size of sample, light cured for 40 s on both sides) of each orthodontic adhesives), as well as the shear bond strength in Mega Pascal unit (MPa) were collected and exposed to one-way analysis of variance (ANOVA) and Tukey’s post-hoc tests.

**Results:**

orthodontic adhesive with 1% weight ZrO2-TiO2 showed the highest mean compressive (73.42 ± 1.55 MPa, p: 0.003, F: 12.74), tensile strength (8.65 ± 0.74 MPa, p: 0.001, F: 68.20), and shear bond strength (20.05 ± 0.2 MPa, p: 0.001, F: 0.17).

**Conclusions:**

Adding ZrO2-TiO2 nanoparticle to orthodontic adhesive increased compressive strength, tensile strength, and shear bond strength in vitro, but in vivo studies and randomized clinical trials are needed to validate the present findings.

## Background

Failure in the bonding system of orthodontic brackets will lead to frequent debonding of orthodontic brackets that delays treatment results. Several tooth- or material-related factors can affect the bonding systems and the failure rates of orthodontic brackets; about 5–7% of clinical bond failures occur for different reasons [1, 2].

Shear bond strength of orthodontic composite resin is greater than resin-modified glass-ionomer cement and polyacid-modified composite resin [3]. Other studies show that shear bond strength was not significantly different when tested over a period of time after orthodontic brackets bonding [4].

In order to improve the properties of resin-based composite, previous studies have focused on the pretreatment of inorganic fillers [5, 6], resin monomers [7], and the development of curing methods. Heat-curing and post-curing heat treatments both increase the degree of polymerization and improve the composite strength to some extent [8]. Nanofillers and fibers have been proposed as reinforcing fillers in dental composite, adding these fillers and fibers will result in increase in composite strength [9].

With the great development of nanotechnology and Nano-phased materials, much attention is directed toward the use of Nano-sized fillers to reinforce the denture base resins, thus producing a polymer nanocomposite with improved mechanical and physical properties as compared to those filled with micro-scale particles; furthermore, the use of multiple Nano fillers rather than a single additive develops a high performance composite that cannot be achieved by using a single filler [10]. The mechanical properties of the resultant polymer nanocomposite depend strongly on the dispersion and adhesion of the filler at the filler matrix interface, thus surface treatment of the fillers with a silane coupling agent is necessary to improve compatibility between the filler and matrix [11].

Both Zirconium oxide (ZrO_2_) and Titanium dioxide (TiO_2_) nanoparticles have interesting mechanical, physical, and photocatalystic properties that make them suitable additives; furthermore, many properties of these mixed nanostructured-metal oxides (ZrO_2_:TiO_2_) were reported to be better than single additives mainly due to the size difference between titanium and zirconium [12].

Several antibacterial components have been added to dental composite resin and adhesive system to disinfect the area around the adhesive restorations for a prolonged duration such as dodecylamine, bipyridine, tannic acid derivatives, polyhexanide, amphilic lipids, silver, Chlorhexide gluconate and fluorides, also incorporating TiO_2_ to orthodontic adhesive can enhance antibacterial effect and decrease surface roughness of orthodontic adhesives [13].

The objective of this study was to evaluate the effect of incorporation of ZrO_2_–TiO_2_ Nano fillers which can have antibacterial effect on mechanical properties of orthodontic adhesive; compressive strength, tensile strength, and shear bond strength.

The null hypothesis was that there was a statistically significant deference between mean values of compressive, tensile and shears bond strengths in orthodontic adhesive after adding ZrO_2_–TiO_2_ Nano fillers in deferent concentrations.

## Methods

All materials that been used in the study with manufacturer information are listed in Table 1.

**Table 1:** Materials

	Materials	Trade	Manufacturer
1	Zirconium oxide (ZrO2) nanofiller 70–80 nm	HWNANO	China
2	Titanium oxide (TiO2) Nanofiller < 50 nm.	Nanoshell	USA
3	Trimethoxysilylpropyl methacrylate 98%Silane	No 2530- 85-8.	Sigma- Aldrich (Germany)
4	Orthodontic Adhesive	Transbond XT, Adhesive composite 3 M Unitek, Monrovia	USA

### Surface modification of nano fillers

Introducing the reactive groups onto the surface of Nano fillers was achieved by their action of the trimethoxysilylpropyl methacrylate (TMSPM) silane coupling agent with the Nano fillers using 5% weight (wt.) of TMSPM for surface modifications of ZrO_2_ nanoparticles and 75% wt. of TMSPM for surface modifications of TiO_2_ nanofillers [14]. ZrO_2_ and TiO_2_ (1:1) Nano fillers by weight were added into dental adhesive used in orthodontics with different concentrations (1 and 0.5% respectively).

### Preparation of modified adhesives

To achieve dental adhesives containing 10% nanoparticles, 64 mg nanopowder was blended into 576 mg (Transbond™ XT Light Cure Adhesive composite, 3 M Unitek), using a mixing spatula on a glass slab in a semi-dark environment until a uniform consistency was achieved. Two hundred mg of the 10% wt. blended composite was then mixed with 200 mg of the original composite to obtain 0.5% wt. containing composite, similarly, 40 mg of 10% composite was blended with 360 mg original composite for the 1% w/w composite [15]. After mixing the particles with composite the resultant composite is passed through two roll mills for about five minutes in a mastication stage, for better distribution of nanoparticles in the matrix [16].

### Grouping of the specimens

**Fig. 1:**
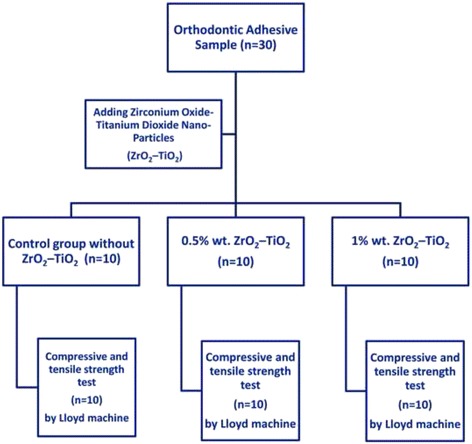
Flow diagram of specimens groups for compressive and tensile strength tests

**Fig. 2:**
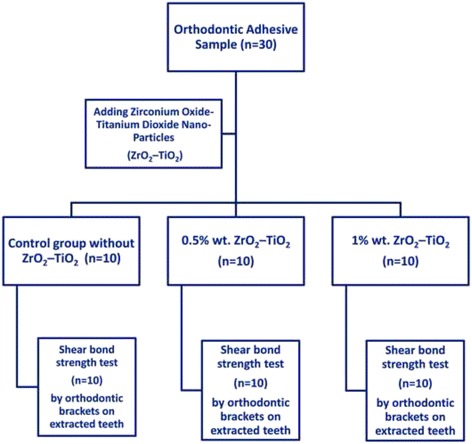
Flow diagram of specimens groups for shear bond strength test

A total of 60 specimens were used in this study. The specimens of orthodontic adhesive were divided into three main groups (20 specimens each) according to the percentage of ZrO_2_–TiO_2_ nanopowder that was added to the dental adhesive with concentrations 0.5%, 1% and control group without any additive. Every group was further subdivided into two subgroups (10 specimens each) according to the type of test as shown in Table 2, Figs. 1 and 2.

**Table 2:** Orthodontic adhesive groups

Groups	Group description	Type of test	Quantity
Group A	Orthodontic adhesive without any additives (control group)	-Compressive and tensile strength	-10 specimens
		- Shear bond strength	-10 specimens
Group B	Orthodontic adhesive with 0.5% wt.	-Compressive and tensile strength	-10 specimens
	ZrO2-TiO2 nanofillers.	- Shear bond strength	-10 specimens
Group C	Orthodontic adhesive with 1% wt.	-Compressive and tensile strength	-10 specimens
	ZrO2-TiO2 nanofillers	- Shear bond strength	-10 specimens
Total			60 specimens

### Compressive strength

#### Mold construction

4 mm diameter by 6 mm height cylindrical Teflon mold was fabricated according to the International Standards Organization (ISO) No. 9917 (2000).

#### Specimens preparation

The orthodontic Adhesive was condensed in the Teflon mold, which was then placed on a glass plate according to each group. Specimens were covered with celluloid strips and pressed with another glass plate. All of the specimens were exposed to light cure for 40 s from both sides (BG-light-LTD, 4002 Plovdiv, 430-490 nm, Bulgaria). The specimens were stored in distilled water for 24 h prior to testing. Curing radiometer equipment (LI-189.Li-Cor Inc, Lincoln, NE68504, USA) was used to ensure steady light intensity throughout the polymerization of all specimens.

#### Compressive strength testing

Specimens were loaded on the Lloyd mechanical testing machine (model LRX plus II, Fareham, England) at crosshead speed of 0.5 mm/min. The specimens were placed with flat ends vertically laid between the two metal plates.

The load was applied until the specimen was crushed; the peak force required to fracture each specimen was recorded in Newton from the stress-strain curve. The compressive strength was calculated in Mega Pascal (MPa) using the following equation:

$$ \mathrm{Compressive}\ \mathrm{Strength} = 4\mathrm{P}/\uppi \mathrm{d} $$

Where (P) is the load at the fracture point in Newtons and d is the diameter of the specimen.

#### Diametric tensile strength

Measuring the tensile strength of orthodontic adhesive is done using an indirect tensile test, a compressive load is placed on the diameter of a cylindrical specimen. The compressive stress induces a tensile stress in the plane of the application of the force. The tensile stress is directly proportional to the compressive load.

In this test, the specimen cylinder was mounted on the Lloyd mechanical testing machine and the load was applied to the specimen using a crosshead speed of 0.5 mm/min applying a compressive force on the specimen until fracture. The diametric tensile strength was calculated in (MPa) using the following equation:

$$ \mathrm{Diametric}\ \mathrm{Tensile}\ \mathrm{Strength} = 2\mathrm{P}/\uppi \mathrm{d}\mathrm{t} $$

Where (P) is the load at the fracture point in Newtons, (d) is the diameter of the specimen, and (t) is the thickness of the specimen.

#### Shear bond strength

Thirty freshly extracted, human maxillary first premolars for orthodontic reasons were collected and stored in a solution of 0.1% thymol. The criteria for tooth selection included intact buccal enamel, not subjected to any pretreatment chemical agents, no cracks, and no caries [17]. The teeth were placed and mounted in metal rings using cold-cure acrylic resin and numbered randomly; then the teeth were polished for 10 s with non-fluoridated pumice using prophylactic rubber cups [18–20].

Metal brackets (Dentaurum®, Discovery®) of maxillary first premolars with an average base surface area of 10.5 mm^2^ were used in this study. Then the teeth were randomly divided into three groups, each consisting of 10 teeth, and bonded as shown in Table 2 (groups A, B and C).

The groups were bonded using 3 M Unitek; Transbond™ Plus Self-Etching Primer one bottle, which was applied for 20 s then dried with mild air flow, then light cured for 10 s.

Group A (the control group), the orthodontic brackets bonded to the tooth surface using Transbond™ XT Light Cure Adhesive.

Groups B and C, the orthodontic brackets bonded to the tooth surface using the modified adhesive as described in Table 2.

The specimens were immersed in 37 °C water for 24 h, and then subjected to thermo-cycling to simulate clinical thermal stress conditions before testing. These directions are according to the American National Standards Institute/American Dental Association (ANSI/ADA) [21], and International Organization for Standardization (ISO) [22] for direct filling resins and dental adhesion.

All specimens were subjected to thermo-cycling by being stored alternatively in water reservoirs at 5 °C and 55 °C, respectively, remaining in each reservoir for 30 s [23].

### Shear bond strength testing

Shear bond strength testing was done using a Lloyd universal testing machine. Upper attachment knife-edge was used; Plastic cylinders with embedded teeth and brackets were mounted on a joint (lower attachment) and aligned in the testing apparatus to ensure consistency for the point of force application and direction of the debonding force for all specimens.

The direction of the debonding force was parallel to the enamel surface in an occlusal-gingival direction. The load was applied perpendicular to the interface of the tooth and bracket at a crosshead with speed of 0.5 mm/min until debonding occurred.

The shear bond strength in kg/cm^2^ was calculated based on the following equation:

$$ \mathrm{Shear}\ \mathrm{Bond}\ \mathrm{Strength} = \mathrm{P}/\uppi .{\mathrm{r}}^2 $$

Where (P) is shear load in kilograms, (r) is the radius of the specimen in millimeter.

Then the shear bond strength was converted to MPa by multiplying the results by 0.09807.

The recorded values of compressive, tensile, and shear bond strength in (MPa) were collected, and statistically analyzed. One-way analysis of variance (ANOVA) and Tukey’s tests were used for testing the significance between the means of tested properties of all tested materials. This was statistically significant when the *P* value ≤ 0.05.

## Results

The comparison between mean compressive strengths in (MPa) is shown in Table 3, comparison between mean tensile strengths in (MPa) is shown in Table 3, and comparison between mean shear bond strengths in (MPa) is shown in Table 4.

**Table 3:** Comparison between mean compressive and tensile strength in (MPa) of adhesive groups

Groups	Nanoparticle percent	N	Range	Mean (SD)	95% CI	F	*p*-value	Tukey post Hoc
Compressive strength								
Group A	without nanofillers	10	53.65 – 57.23	54.92 (4.15)	53.6–55.3	12.74	0.003	Group C < Group A, Group B < Group A
Group B	0.5 wt %	10	65.96 – 72.68	69.28 (3.76)	68.0–70.5			
Group C	1 wt %	10	69.33 – 76.58	73.42 (1.55)	71.7–74.2			
Tensile strength								
Group A	without nanofillers	10	2.21 – 6.38	4.92 (0.54)	4.1–5.7	68.20	0.001	Group C < Group A
Group B	0.5 wt %	10	6.54 – 8.35	6.14 (0.71)	5.1–6.5			
Group C	1 wt %	10	7.42 – 9.64	8.65 (0.74)	7.8–8.5			

*N* Number of sample, *CI* Confidence interval, *SD* Standard deviation

**Table 4:** Comparison between mean shear bond strength in (MPa) of adhesive groups

S hear bond strength								
Groups	Nanoparticle percent	N	Range	Mean (SD)	95% CI	F	*p*-value	Tukey Post Hoc
Group A	without nanofillers	10	13.21 – 16.79	14.75 (0.25)	13.6–15.1	0.17	0.001	Group C < Group A, Group B < Group A
Group B	0.5 wt %	10	18.34 – 22.41	20.32 (0.47)	19.4–20.9			
Group C	1 wt %	10	23.22 – 27.58	25.05 (0.2)	14.3–20.3			

*N* Numb er of sample, *CI* Confide nce interval, *SD* S tandard deviat ion

All groups of orthodontic adhesive with nanoparticles (B and C) showed compressive strength, tensile strength, and shear bond strength values higher than that of the control group (A).

The power analysis revealed that a total sample size of 30 (10 per group) was needed to detect clinically meaningful differences between the groups at a power of 85% and at 0.05 significance level.

### Compressive and tensile strengths

Orthodontic adhesive specimens with ZrO_2_–TiO_2_ Nano fillers (groups C and B) showed significantly high compressive and tensile strengths when compared with the control group, while the increase in compressive and tensile strengths was not significant between Group C (1% wt. ZrO_2_–TiO_2_) and Group B (0.5% wt. ZrO_2_–TiO_2_).

### Shear strength

Table 4 shows the comparison between the mean shear bond strength of three tested groups. Orthodontic adhesive specimens with 1% wt. ZrO_2_–TiO_2_ Nano fillers (Group C) showed a significantly highest shear bond strength followed by the orthodontic adhesive specimen with 0.5% wt. ZrO_2_-TiO_2_ Nano fillers (Group B), while the control group was significantly lowest shear bond strength.

Therefore the null hypothesis that compares the compressive, tensile and shears bond strengths after adding ZrO_2_–TiO_2_ Nano fillers to orthodontic adhesive was accepted and not rejected in this study.

## Discussion

Several factors, such as failure in the bonding technique, low retentiveness of certain bracket bases, masticatory forces, and reduced size of the bracket base for esthetic reasons, can result in bracket debonding [24, 25] that will prolong the treatment time and increase the treatment costs. Several solutions had been proposed, such as aluminum-oxide sand blasting and primer application [26], to minimize the unwanted debonding rates in this study by adding ZrO_2_–TiO_2_ to orthodontic adhesive. The results showed an increase in the shear bond strength (20.32 and 25.05 MPa), although 5.9 to 7.5 MPa is the minimum acceptable shear bond strength for routine clinic use, as considered by Reynolds [27], we don’t always have an ideal situation for bonding brackets and up to 21 MPa shear bond strength is acceptable for orthodontic treatment. The failure rate of brackets with Transbond™ Plus range from 0.94% to 7.4% [28–31].

In this study, the self-etching primer had been applied for 20 s prior to the process of light curing (unlike the manufacturer’s instructions) which would yield superior performance of the etching primer. This was done by prolonging the application time, as shown by dos Santos,et al [29].

In order to simulate the mastication forces that create stresses to the restoration materials a compressive strength test are used in laboratory experiments [32]. Most mastication forces are not constant and the exact values are not known [33].

Measuring the shear bond strength for bonded orthodontic brackets is very common in literatures, it is a simple test when compared to tensile bond strength tests, in which it is difficult to align the specimen in the testing machine without creating deleterious stress distributions [34, 35].

Titanium dioxide (TiO_2_) nanofiller has an antibacterial effect, biocompatibility and minimum toxicity. This nanofillers beside their antibacterial effect they have been suggested as reinforcing fillers [36, 37].

Zirconium oxide (ZrO_2_) was used because it has excellent biocompatibility and white color which is less likely to alter its esthetic. The nanofiller particles were used in this study as they yield a better dispersion, eliminate aggregation, and improve its compatibility with organic polymer [38].

Both ZrO_2_ and TiO_2_ nanoparticles were reported to be better than single additives mainly due to the size differences between titanium and zirconium [12]. In this study, compressive, tensile, and shear bond strength improvement of orthodontic adhesive containing 1% and 0.5% (w/w) ZrO_2_–TiO_2_ nanoparticles can be attributed to the small sizes of the ZrO_2_ and TiO_2_ and also to the surface modification of the nanofillers with TMSPM coupling agents which provide better dispersion of particles in matrix, avoid agglomeration, and improves interfacial adhesion of the fillers to the polymer matrix [39].

Shear bond strength can be 40% less when measured in vivo than in vitro and this makes our results in shear bond strength higher than what will be in real clinical cases, adding ZrO_2_–TiO_2_ nanoparticles can enhance the shear bond strength for optimum clinical use, further in vivo studies are needed [40].

Color stability, polymerization shrinkage, antibacterial effect and toxicity of adding ZrO_2_–TiO_2_ nanoparticles on resin-based adhesives could be studied in the future with other mechanical and physical properties in different concentrations of Nano filler.

## Conclusions

On the basis of this study, we can conclude that the addition of ZrO_2_–TiO_2_ nanoparticles on resin-based adhesives increase the compressive, tensile, and shear bond strengths of the adhesive in vitro.

## Abbreviations

MPa: Mega pascal
N: Newton
RBC: Resin-based composite
TiO_2_: Titanium dioxide
Wt.: Weight
ZrO_2_: Zirconium oxide
ZrO_2_–TiO_2_: Zirconium oxide–titanium dioxide
